# Structural and Functional Characteristics of Soil Microbial Communities in Forest–Wetland Ecotones: A Case Study of the Lesser Khingan Mountains

**DOI:** 10.3390/life15040570

**Published:** 2025-04-01

**Authors:** Junnan Ding, Shaopeng Yu

**Affiliations:** Heilongjiang Province Key Laboratory of Cold Region Wetland Ecology and Environment Research, Harbin University, Harbin 150086, China; wetlands1972@126.com

**Keywords:** bacterial diversity, high-throughput sequencing, soil microorganisms, soil physicochemical properties, vegetation types

## Abstract

Soil microorganisms play an essential role in vegetation succession, nutrient cycling, and ecosystem restoration. This study investigates the responses of soil microbial communities to ecological transitions from forest to wetland in the Lesser Khingan Mountains, including mixed forest, conifer forest, wetland edge, and natural wetland. The results indicated that natural wetland soils were weakly acidic and contained significantly higher organic matter, total nitrogen, and available phosphorus compared to other soils. Soil bulk density increased with depth. Actinobacteria, Acidobacteriota, and Proteobacteria dominated in mixed forest, wetland edge, and natural wetland soils, respectively, showing minimal variation between depths. Principal component analysis and non-metric multidimensional scaling demonstrated distinct bacterial communities between natural wetlands and wetland edges. Redundancy analysis revealed that soil bacterial communities differed significantly between 15 cm and 30 cm layers, influenced by potassium, bulk density, organic carbon, phosphorus, and nitrogen. Proteobacteria and Bacteroidota abundances correlated positively with nutrients, while Acidobacteriota and Verrucomicrobiota correlated negatively with available potassium. Chemotrophic and aerobic bacteria dominated in forest soils, whereas fermentation-related and anaerobic bacteria were prevalent in wetland soils. The study highlights how ecological transitions and soil properties shape soil microbial communities and their functions.

## 1. Introduction

The Lesser Khingan Mountains are one of the most extensive distribution areas of forest wetlands in China, characterized by rich plant resources and minimal human disturbance. Forest wetlands are forest plant communities dominated by hygrophilous and marsh plants, occurring in areas with excessive surface moisture or standing water, and they play a crucial role in wetland ecosystems [1]. They are irreplaceable in terms of greenhouse gas emissions, water conservation, soil retention, and biodiversity maintenance [2]. In recent years, global climate change has led to a continuous increase in temperature and a rise in groundwater levels in the Lesser Khingan Mountains, resulting in the degradation of forest wetland ecological functions [3,4]. Soil microorganisms, as key mediators of nutrient mobilization and energy flow, are highly sensitive to environmental changes, such as vegetation, climate, and land use [4]. There are few reports on the impact of soil microbial species diversity in the ecotone between forest and grassland ecosystems, making this a promising emerging focus in ecological and microbiological research [5]. Investigating the soil microbial community structure in the forest wetlands of the Lesser Khingan Mountains is essential not only for understanding soil quality changes but also for providing scientific insights into the restoration and conservation of wetland ecosystems.

Ecotones are transitional zones characterized by discontinuities in biological and environmental attributes within ecosystems [6]. They are widely distributed across various spatial scales, ranging from ecological regions and biomes to communities and even finer scales [7]. The forest–wetland ecotone can be broadly categorized into two types: the mountain vertical forest–wetland ecotone, primarily shaped by elevation gradients, and the flatland horizontal forest–wetland ecotone, influenced mainly by climatic gradient variations [8]. Forest–wetland ecotones are noted for their high environmental heterogeneity and strong sensitivity to global climate change [9]. Their unique habitat conditions foster endemic or edge species, creating complex plant community structures that enhance species coexistence [10,11]. These ecotones exhibit weak resistance to disturbances, pronounced edge effects, and considerable environmental variability, making them particularly responsive to climate and land use changes [12]. Previous studies on forest–wetland ecotones have predominantly focused on plant community diversity and distribution patterns, often overlooking the roles and dynamics of soil microbial communities [13]. Additionally, earlier research has rarely accounted for variations in microbial functions across different soil depths, resulting in an incomplete understanding of microbial diversity and functional specialization within these ecosystems [14]. Moreover, the environmental complexity of forest–wetland ecotones, such as variations in moisture availability, organic matter accumulation, and fluctuating nutrient inputs, plays a crucial role in shaping microbial community composition and functional attributes, yet this relationship remains insufficiently explored [15]. Considering these limitations, there is a clear necessity to comprehensively investigate soil microbial community structures and their functional specificity, including the influence of both soil depth variation and ecotone-driven environmental factors [16]. Microbial functional traits, such as nutrient cycling capabilities, organic matter decomposition, and stress tolerance mechanisms, play a vital role in maintaining ecosystem stability in forest–wetland ecotones [17]. For example, microbial-mediated nitrogen and carbon cycling regulate soil fertility and productivity, while organic matter decomposition contributes to nutrient turnover and availability [18]. Furthermore, microbial resilience to environmental fluctuations enhances ecosystem adaptability to climate change and land use transformations [19]. These functional traits collectively support ecosystem stability, promote efficient nutrient cycling, and enhance the resilience of forest–wetland ecotones against environmental stressors [20]. Plant diversity in forest–wetland ecotones is influenced by various factors, such as topography, nutrient cycling, and global environmental changes [21]. In contrast, microbial diversity is shaped by soil depth, litter decomposition, climate change, soil organic matter content, and land use types [13]. Given these ecological dynamics, employing advanced theoretical frameworks and technological approaches to investigate plant–microbe interactions, soil microbial community structures, and functional specialization in forest–wetland ecotones is highly significant [22]. This research will not only enhance our understanding of microbial adaptation mechanisms under varying environmental conditions but will also elucidate soil microbial diversity dynamics and ecosystem functionality and stability responses [23]. Ultimately, these findings will provide a scientific basis for maintaining ecosystem health and stability in forest–wetland ecotones, thereby contributing to effective conservation and ecological management strategies for these critical transitional zones [24]. High-throughput sequencing, including 16S rRNA gene sequencing and metagenomic analysis, will be utilized to investigate microbial community composition and functional potential. These methods enable the identification of unculturable microorganisms, the detection of functional genes, and a comprehensive understanding of microbial interactions within the forest–wetland ecotone, providing deeper insights compared to traditional culture-based techniques.

Despite the ecological significance of forest–wetland ecotones, research on their microbial communities remains limited, particularly concerning the functional traits that drive ecosystem processes. Key microbial functions, such as biofilm formation, nitrogen fixation, and metal tolerance, play a crucial role in maintaining soil stability, regulating biogeochemical cycles, and facilitating plant–microbe interactions. However, the extent to which these functional traits vary across different ecotones and their specific contributions to ecosystem dynamics remain poorly understood. Addressing these knowledge gaps is essential for advancing our understanding of microbial functional diversity in transitional environments. The overall goal of this research is to elucidate how soil physicochemical properties across forest–wetland ecotones influence microbial community composition and their functional roles in ecosystem processes. Specifically, the objectives of this study are as follows: (1) to explore how variations in soil physicochemical properties across different forest–wetland ecotones influence microbial community composition, particularly with changes in soil depth; (2) to analyze the differences in functional traits, such as biofilm formation, nitrogen fixation, and metal tolerance, across soil bacterial communities in different ecological ecotones and assess their role in ecosystem processes; and (3) to investigate how nutrient concentrations, such as organic carbon, nitrogen, potassium, and phosphorus affect the functional traits of soil bacterial communities. This study will contribute to a deeper understanding of microbial community structure and function in soils across different ecological ecotones, providing theoretical insights for ecosystem management and soil health maintenance.

## 2. Materials and Methods

### 2.1. Site Description

**Figure 1:**
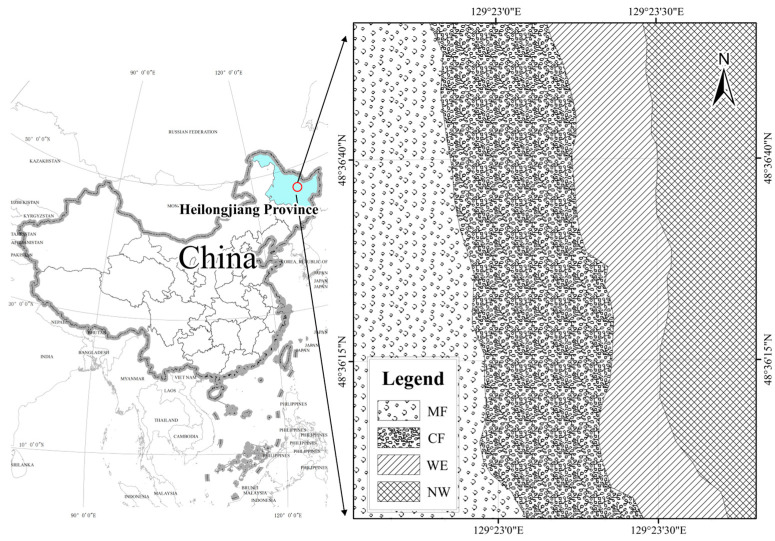
Map showing different vegetation location of the study. Abbreviations: mixed forest (MF), conifer forest (CF), wetland edge (WE), and natural wetland (NW).

The study site is located in the Wuyiling Forestry Bureau of the Lesser Khingan Mountains (48°33′–48°50′ N, 129°00′–129°30′ E), a region characterized by an extensive distribution of rivers, lakes, and marshes. As one of the most well-preserved wetlands in China’s high-latitude forest regions, it provides a crucial habitat for diverse flora and fauna. The area experiences a temperate continental monsoon climate, with an average annual temperature of −1.1 °C, a frost-free period of 97 days, and an average annual precipitation of 584 mm, most of which occurs between July and August. The dominant soil types are peat soil and gley soil. Along the environmental gradient from swamp to forest, three typical transition zones were identified for this study: swamp wetland, conifer forest, and mixed forest (Table 1, Figure 1). To comprehensively capture the forest–wetland transition, this study selected ecotones that represent distinct vegetation composition, hydrological conditions, and soil properties, which are key factors influencing microbial diversity and functional adaptations.

### 2.2. Sample Collection

In September 2024, within each designated ecotone plot, three quadrats were randomly established, each covering an area of 40 m^2^, with a spacing of approximately 50 m between plots. The selection of three quadrats per ecotone was based on the need to balance spatial representation and practical feasibility, ensuring sufficient replication for statistical reliability in microbial diversity analysis. Within each quadrat, 10 observation subplots (1 m × 1 m each) were arranged in an S-shaped pattern to ensure spatial randomness and to capture microhabitat variability. A total of 72 soil samples were collected. Soil samples were taken at two depths (0–15 cm and 15–30 cm) following the natural soil profile sampling method. During sampling, plant roots, litter, stones, and other impurities were removed. After collection, soil samples from the same plot were thoroughly mixed, bagged, and labeled accordingly. To maintain sample integrity, field soil samples were stored in an ice box at approximately 4 °C and immediately transported to the laboratory. In the lab, a portion of fresh soil (~10 g) was transferred into a sterile 50 mL Falcon tube and stored at −80 °C for microbial analysis. The remaining soil was air-dried, sieved (2 mm), and homogenized for physicochemical analysis. Soil properties, including pH and nutrient content, were measured after sample processing to ensure consistency and comparability across all samples.

### 2.3. Analysis of Soil Physicochemical Properties

The physicochemical properties of the soil samples were analyzed using standard laboratory procedures with detailed methodological specifications. Soil pH was measured using a pH meter (Mettler Toledo SevenCompact S220, Mettler Toledo, Greifensee, Switzerland), calibrated with standard buffer solutions (pH 4.0, 7.0, and 10.0). Soil organic carbon (SOC) content was determined by the Walkley–Black titration method [25]. The soil bulk density (BD) was measured using the core method, where soil samples of a fixed volume were collected with a core sampler and oven-dried at 105 °C to a constant weight; the dry mass was then divided by the core volume to calculate bulk density [18]. Total nitrogen (TN) was quantified using the Kjeldahl method following concentrated H_2_SO_4_ digestion [26]. Alkali-hydrolyzable nitrogen (AN) was analyzed using the alkali-hydrolyzable diffusion method [27]. Total phosphorus (TP) content was determined via HClO_4_-H_2_SO_4_ digestion followed by molybdenum–antimony colorimetry, while available phosphorus (AP) was extracted with NaHCO_3_ and quantified using the same colorimetric method [28]. Total potassium (TK) was measured by digesting the soil samples with a mixture of nitric acid (HNO_3_), perchloric acid (HClO_4_), and hydrofluoric acid, followed by analysis using flame photometry or atomic absorption spectrophotometry (AAS) (PerkinElmer AAnalyst 400, PerkinElmer, Waltham, MA, USA) [29]. Available potassium (AK) was extracted using 1 M ammonium acetate (pH 7.0) and quantified via flame photometry (Sherwood Model 420, Sherwood Scientific Ltd., Cambridge, UK) or AAS (PerkinElmer AAnalyst 400) [30]. Detection limits for spectrophotometric methods were set according to manufacturer specifications to ensure analytical precision.

### 2.4. DNA Extraction and High-Throughput 16S rRNA Gene Paired-End Sequencing

Genomic DNA was extracted from 0.5 g of soil using the Omega E.Z.N.A.^®^ Soil DNA Kit (Omega Bio-Tek, Norcross, GA, USA) following the manufacturer’s protocol. The quality and purity of the extracted DNA were assessed using a NanoDrop 2000 spectrophotometer (Thermo Fisher Scientific, Waltham, MA, USA), measuring A260/A280 ratios to ensure DNA purity. Additionally, agarose gel electrophoresis (1% *w/v*) was performed to check the DNA integrity. A two-step polymerase chain reaction (PCR) was carried out on a GeneAmp 9700 PCR system (Applied Biosystems, Thermo Fisher Scientific, Waltham, MA, USA). In the first step, universal primers 515F (5′-GTGCCAGCMGCCGCGGTAA-3′) and 907R (5′-CCGTCAATTCMTTTRAGTTT-3′) were used to amplify the V3–V4 region of the bacterial 16S rRNA gene. In the second step, barcodes were added. The PCR mixture (25 μL) contained 1× PCR buffer, 1.5 mM MgCl_2_, 0.2 mM dNTPs, 0.5 μM of each primer, 1.25 U of Taq DNA polymerase (Takara, Japan), and 1 μL of template DNA. PCR conditions included an initial denaturation at 95 °C for 3 min, followed by 25 cycles of denaturation at 95 °C for 30 s, annealing at 55 °C for 30 s, and extension at 72 °C for 45 s, with a final extension at 72 °C for 10 min. To assess potential PCR inhibitors, a dilution series was performed for a subset of DNA extracts, ensuring that PCR amplification efficiency was not compromised. The PCR product was purified using a PCR cleanup kit (Omega Bio-Tek) and quantified with a QuantiFluor^®^-ST fluorometer (Promega, Madison, WI, USA) before being adjusted for sequencing. The samples were then sent to Shanghai Meiji Biotechnology Co., Ltd. (Shanghai, China) for sequencing on the Illumina HiSeq 2500 PE250 platform (San Diego, CA, USA) for high-throughput sequencing [31].

### 2.5. Sequencing Data Processing and Analysis

Sequencing data were processed using QIIME (Caporaso Lab, Northern Arizona University, Flagstaff, AZ, USA) and DADA2 (Benjamin Callahan, North Carolina State University, Raleigh, NC, USA). Quality filtering was performed by removing sequences with a quality score below Q20 and those shorter than 200 bp. Chimeric sequences were detected and removed using the UCHIME algorithm. Adapter sequences were trimmed using Cutadapt (version 3.4), and non-bacterial sequences were identified and excluded based on alignment with the SILVA database (version 138). Singletons (OTUs appearing only once across all samples) were filtered out to minimize sequencing artifacts.

### 2.6. Statistical Analysis

Community diversity parameters (Shannon, Sobs, Ace, and Chao1 indices) were used to conduct alpha diversity analyses using the mothur software (version 1.44.3) [32]. Beta diversity analysis was performed using R software (version 4.2.2; R Core Team, Vienna, Austria), and differences in microbial communities were assessed using one-way analysis of variance (ANOVA) and the least significant difference test. Data were statistically analyzed using Microsoft Excel 2007 (Redmond, WA, USA) and SPSS 22.0 (IBM, Inc., Armonk, NY, USA). Microbial functions of the soil bacteria were predicted by FAPROTAX and FUN Guide [33,34]. BugBase (Bengaluru, India) was used to annotate the functions of bacteria, and the OTU table clustered by 97% sequence similarity was used as the input file. The output table was standardized by the predicted number of 16S copies, and the microbial phenotype was then predicted using the preprocessed database. The threshold was automatically selected by the BugBase tools, categorizing bacteria into groups, such as aerobic, anaerobic, stress-tolerant, Gram-negative, Gram-positive, and potentially pathogenic [35,36].

## 3. Results

### 3.1. Soil Physicochemical Properties

There were significant differences in soil physicochemical properties across different ecological ecotones (Table 2). Soil pH ranged from 5.38 (MF_30_) to 6.07 (WE_30_), indicating a generally weakly acidic condition, with natural wetland soils exhibiting significantly higher pH values than mixed forest soils (*p* < 0.05). The contents of SOC, TN, AN, AP, and AK varied notably across different ecotone types. Within both the 0–15 cm and 15–30 cm soil layers, SOC, TN, AN, AP, and AK concentrations in NW soils were significantly higher than those in WE, MF, and CF soils (*p* < 0.05). Soil bulk density in the 15–30 cm layer was consistently higher than that in the 0–15 cm layer (*p* < 0.05). Additionally, TN, AN, AP, and AK concentrations showed a decreasing trend with increasing soil depth. No significant differences were observed in TK and TP concentrations between CF_15_ and CF_30_.

### 3.2. Analysis of Soil Bacterial Structure in Different Forest–Wetland Ecotones

**Figure 2:**
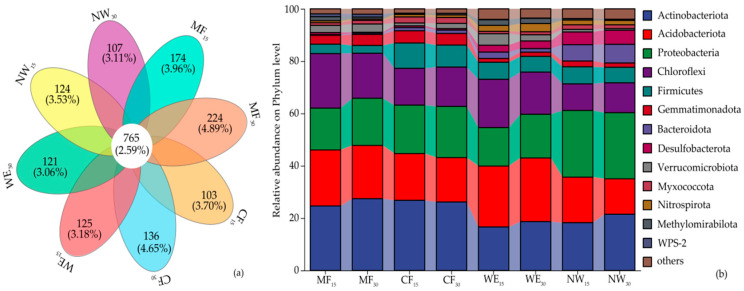
Relative abundance of soil bacterial communities under different forest–wetland ecotones at the level of phylum. (**a**) Venn diagram of OTU-based microbial community. (**b**) Microbial community composition at the phylum level.

As shown in Figure 2a, after rarefying to the minimum sequence depth, taxonomic analysis of OTU representative sequences at a 97% similarity threshold identified a total of 29,514 bacterial OTUs across the four forest–wetland ecotone types. A total of 765 bacterial OTUs were shared among all soil samples. The number of unique bacterial OTUs in each sample was as follows: MF_15_ (174), MF_30_ (224), CF_15_ (103), CF_30_ (136), WE_15_ (125), WE_30_ (121), NW_15_ (124), and NW_30_ (107). Overall, the number of bacterial OTUs in forest ecosystems (MF + CF) exceeded that in wetland ecosystems (WE + NW), and within forest ecosystems, bacterial OTU abundant increased with soil depth (MF_30_ > MF_15_, CF_30_ > CF_15_).

An analysis of the dominant microbial phyla across different ecotones revealed that the major bacterial phyla were largely consistent among the four ecotone soil types (Figure 2b). The predominant bacterial phyla included Actinobacteria, Acidobacteriota, Proteobacteria, Chloroflexi, and Firmicutes. Notably, Actinobacteria exhibited higher relative abundance in MF and CF soils, accounting for 24.65–27.43% and 26.19–26.84%, respectively. In contrast, Acidobacteriota was more abundant in WE soils, with a relative abundance of 23.25–24.31%, while Proteobacteria was predominant in NW soils, with a relative abundance of 25.29–25.49%. Furthermore, microbial community composition at the phylum level showed no significant differences between the 0–15 cm and 15–30 cm soil layers across all soil samples.

### 3.3. Microbial Alpha and Beta Diversity

The alpha diversity index of soil bacteria differed significantly among the four forest–wetland ecotone types (Table 3). The bacterial Shannon diversity index ranged from 5.56 (MF_15_) to 6.40 (NW_15_), but there was no significant difference in the Shannon index among different soil layers within the same soil type. The Chao1 index ranged from 1149.4 (MF_15_) to 1852.32 (NW_30_), with a significantly higher Chao1 index in natural wetlands compared to MF, CF, and WE (*p* < 0.05); however, no significant difference was observed in the Chao1 index among different soil layers within the same soil type. The bacterial Sobs diversity index ranged from 1032.7 (MF_15_) to 1651 (NW_30_), but no significant difference was detected in the Sobs index among different soil layers within the same soil type. The bacterial Ace diversity index ranged from 1139.7 (MF_15_) to 1806.1 (NW_30_).

**Figure 3:**
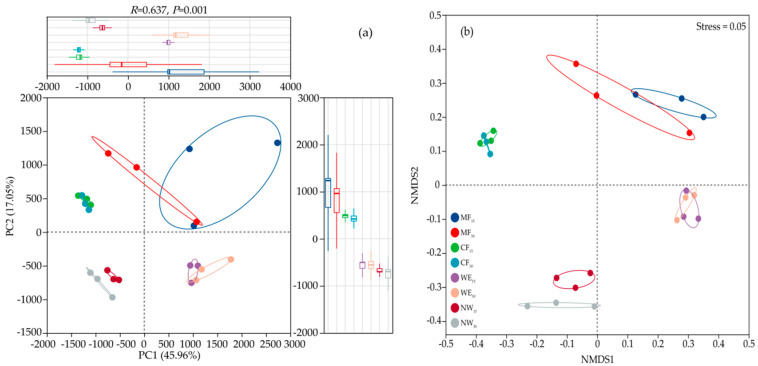
The PCA (**a**) and NMDS (**b**) analysis of soil bacteria in different forest–wetland ecotones.

Principal component analysis (PCA) based on the Bray–Curtis distance algorithm was used to analyze the differences in soil bacterial community composition among the four types of ecotones (Figure 3a). The results showed that at the OTU level, the first two axes (PC1 and PC2) explained 45.96% and 17.05% of the total variation in bacterial species among the ecotone types, respectively, suggesting that soil type reshaped the soil microbiomes (R = 0.637, *p* = 0.001). The NMDS showed a significant difference (*p* < 0.05) in the beta diversity of the bacterial community in different forest–wetland ecotones, and was well-represented (stress = 0.05), as depicted in Figure 3b. Among them, the bacterial communities of NW and NW were farther away from each other in the graph. Thus, the differences between NW and NW were significant. The four forest–wetland ecotones type exhibited variations in the bacterial communities. In the chart, it seems that different soil layers of the same soil type are more similar to each other, indicating that there is little difference in soil bacterial communities between them.

### 3.4. Correlation Between Soil Physical and Chemical Properties and the Relative Abundance of Microbial Communities

**Figure 4:**
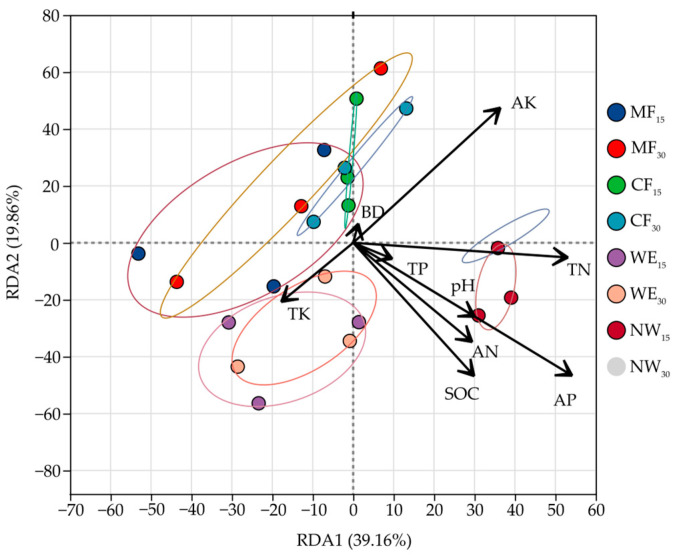
RDA of microbial community composition and soil physicochemical properties.

As shown in Figure 4, the RDA analysis reveals significant differences in soil bacterial communities between the 15 cm and 30 cm soil layers across different ecotone types, influenced by environmental factors, such as potassium, soil bulk density, organic carbon, phosphorus, and nitrogen. The variation in soil depth affects the structure of soil bacterial communities, reflecting the differential impact of environmental factors on bacterial community composition at different soil depths. The first two axes, RDA1 (39.16%) and RDA2 (19.86%), together explain 59.02% of the total variance in bacterial community composition. The differences in bacterial communities and environmental factors between the 15 cm and 30 cm layers follow certain patterns. For MF, the changes in bacterial communities between the 15 cm and 30 cm soil layers are small, mainly influenced by AK and BD, both of which are positively correlated with bacterial communities, suggesting that potassium content and soil compaction have a consistent impact on bacterial communities across both soil layers. In CF, the bacterial communities show more significant differences between the 15 cm and 30 cm layers, especially along RDA1, where the bacterial community at 30 cm is strongly influenced by AK and BD, indicating a stronger positive correlation with potassium content and soil compaction. For WE, the bacterial communities at 15 cm and 30 cm are relatively consistent, and are mainly influenced by SOC and AP, suggesting that organic carbon and phosphorus content in wetland soils continuously promote specific bacterial community structures. For NW, significant differences are observed between the 15 cm and 30 cm layers, with the community at 30 cm being strongly influenced by TP and TN, which show a negative correlation, indicating that these soil properties may inhibit the formation of certain bacterial communities in deeper soil layers.

### 3.5. Correlation Analysis of Soil Bacteria with Soil Physical and Chemical Factors

**Figure 5:**
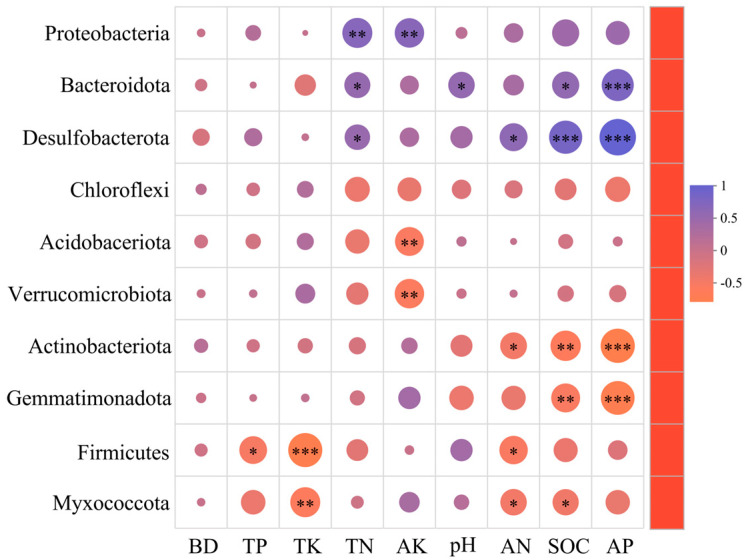
Correlation analysis between the relative abundance of the top 10 bacterial phylum and soil environmental factors. Note: significance levels: * *p* < 0.05, ** *p* < 0.01, *** *p* < 0.001.

As shown in Table 4 and Figure 5, Proteobacteria abundance was positively correlated with TN and AK (*p* < 0.01). Bacteroidota abundance was positively correlated with TN, pH, SOC (*p* < 0.05), and AK (*p* < 0.01), while it was negatively correlated with TK. Desulfobacterota abundance was positively correlated with SOC, AP (*p* < 0.001), TN, and AN (*p* < 0.05). Relative Acidobacteriota and Verrucomicrobiota abundances were negatively correlated with AK (*p* < 0.01). Actinobacteriota and Gemmatimonadota were negatively correlated with SOC and AP (*p* < 0.001). Firmicutes abundance was negatively correlated with TK (*p* < 0.001), AN, and TP (*p* < 0.05). Myxococcota abundance was negatively correlated with TK (*p* < 0.05), AN, and SOC (*p* < 0.05).

### 3.6. Phenotypic Prediction and Functional Analysis

**Figure 6:**
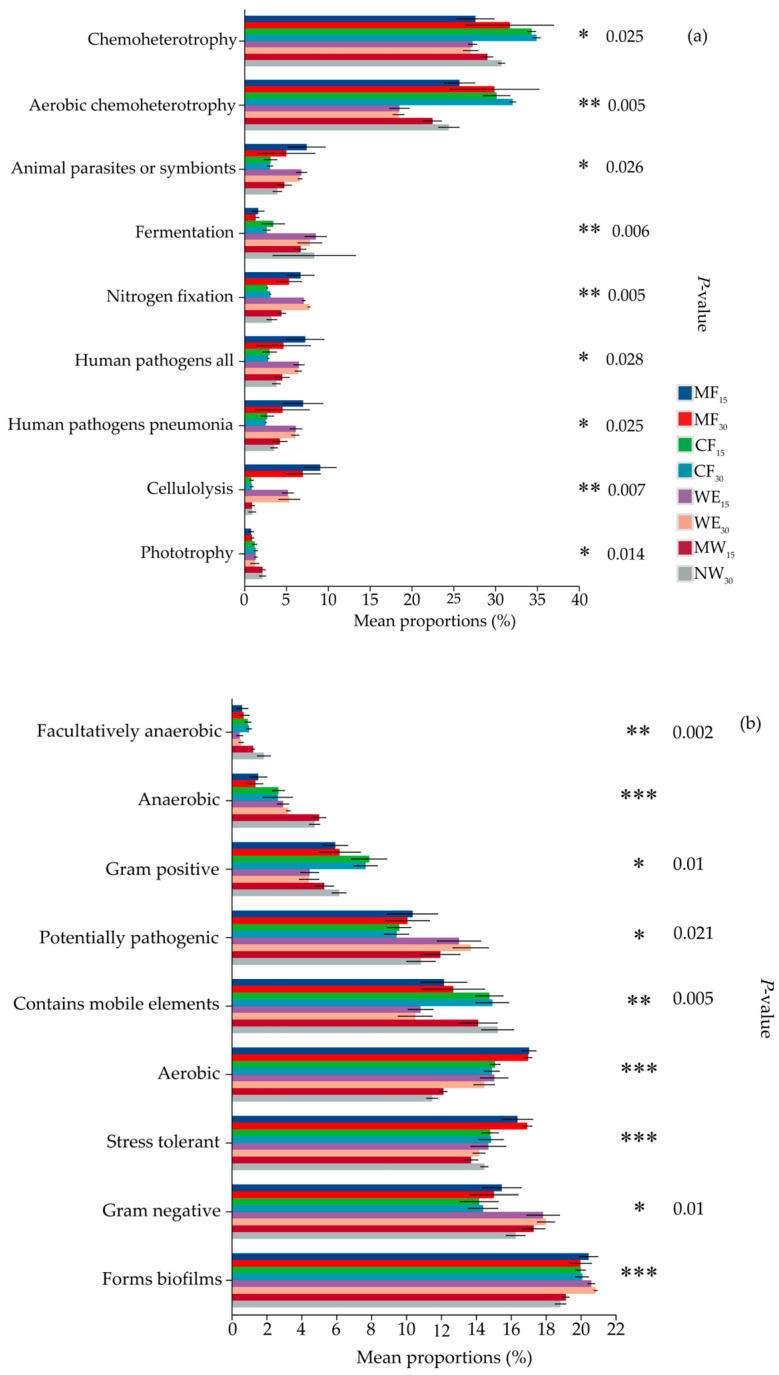
FAPROTAX function prediction (**a**) and BugBase phenotype prediction (**b**) in forest–wetland ecotones type. Note: significance levels: * *p* < 0.05, ** *p* < 0.01, *** *p* < 0.001.

Figure 6a illustrates the functional composition of soil bacterial communities across different ecotone types and soil depths, highlighting significant variations in the abundance of various functional groups. The data reveal distinct differences in the functional composition of bacterial communities across ecotone types and soil depths, reflecting the influence of environmental factors on the functional capabilities of soil bacteria. The abundance of chemotrophic and aerobic chemotrophic bacteria was notably higher in MF_15_, MF_30_, CF_15_, and CF_30_, suggesting that these soil types are characterized by a dominant presence of bacteria relying on chemical compounds and oxygen for energy. In the case of animal parasitic or symbiotic bacteria, MF_15_ exhibited the highest abundance, although no significant differences were observed between MF_30_, WE_15_, and WE_30_. Fermentation-related bacteria were most abundant in WE and NW, indicating more active anaerobic processes in these environments. Nitrogen-fixing bacteria were particularly abundant in MF_15_, MF_30_, WE_15_, and WE_30_, implying that these soils support more efficient nitrogen fixation. The abundance of human pathogenic bacteria was higher in MF_15_, with no significant differences noted compared to other soil depths. The highest abundance of cellulolytic bacteria was found in MF_15_ and MF_30_, suggesting that these soils play a significant role in cellulose degradation.

Figure 6b illustrates the functional traits of soil bacterial communities across different ecotone types and soil depths, revealing significant variations in bacterial abundances. In terms of biofilm formation, NW_30_ exhibited significantly lower abundance compared to other ecotone types (*p* < 0.001), indicating that natural wetland soils at a depth of 30 cm are less conducive to biofilm development. Gram-negative bacteria were most abundant in WE and NW, reflecting a favorable environment for bacteria with a thinner peptidoglycan layer and an outer membrane. The highest abundance of stress-tolerant and aerobic bacteria was observed in MF_15_, suggesting that mixed forest soils at a depth of 15 cm support a higher proportion of bacteria capable of withstanding harsh conditions and relying on oxygen for metabolism. Bacteria that contain metal elements were most abundant in CF_30_ and NW_30_, highlighting the ability of these soils to support metal-tolerant bacteria. The highest abundance of potentially pathogenic bacteria was found in WE, suggesting that wetland soils provide a conducive environment for the growth of pathogenic bacteria. Gram-positive bacteria were most prevalent in MF and CF, reflecting the dominance of bacteria with a thick peptidoglycan layer in these soil types. Anaerobic and facultatively anaerobic bacteria were most abundant in NW, indicating that natural wetland soils favor the growth of bacteria capable of thriving in low-oxygen environments.

## 4. Discussion

### 4.1. Relationship Between Soil Physicochemical Properties and Diversity

Soil pH, carbon, nitrogen, and phosphorus significantly influence soil fertility, plant growth, microbial activity, and nutrient cycling [37]. Variations in these properties across forest–wetland ecotones reveal complex vegetation–soil–microbe interactions [38]. Soils in this study were generally weakly acidic, with wetlands in semi-arid areas displaying near-neutral pH (Table 2). Acidification primarily occurs via the adsorption of H^+^ or Al^3+^ by soil complexes, replacing base cations (Ca^2+^, Mg^2+^, Na^+^, K^+^) due to acidic precipitation [39,40]. Wetland soils’ high organic matter content further contributes to acidification through organic acid production [41] and plant nutrient absorption releasing H^+^ ions [42]. Groundwater fluctuations also influence nutrient leaching in wetlands [43]. Seasonal climatic and hydrological changes alter soil properties and microbial communities, affecting nutrient dynamics [44,45]. The Daxing’anling–Hulunbuir ecotone study indicated acidification and moisture significantly impact plant diversity, notably reducing acid-sensitive species [46]. Thus, monitoring these factors is vital for ecosystem protection and soil health [47]. This study (Table 2) found significantly higher SOC, TN, AN, AP, and AK concentrations in natural wetlands (NW) compared to wetland ecotones (WE), mixed forests (MF), and coniferous forests (CF). High biomass and favorable hydrological conditions in wetlands enhance nutrient accumulation [48]. In contrast, WE, MF, and CF soils have lower nutrients due to weaker organic input and slower nutrient cycling [49]. Nutrient concentrations decreased with soil depth, particularly in NW, reflecting surface plant and organic matter influences [50,51]. Deeper soils (15–30 cm) showed lower nutrients and higher bulk density due to less organic matter and soil compaction [52,53]. Slower nutrient cycling in CF soils resulted in minimal depth-related variations for TK and TP.

Ecotones with marked structural transitions exhibit higher biodiversity than more fluctuating environments [54]. Ecotones near biogeographic boundaries typically show lower alpha diversity but higher beta diversity [55]. Beta diversity studies effectively characterize ecological transition zones [56]. Research in Inner Mongolia demonstrated that stable conditions enhance alpha diversity, while boundary conditions increase beta diversity [57]. Our results (Table 2 and Table 3) confirmed the relationship between soil nutrients, plant communities, and microbial diversity. Wetlands effectively accumulate nutrients, supporting higher microbial diversity due to favorable moisture and biomass [58,59,60]. NW soils exhibited higher bacterial diversity (with a Shannon index of 6.40), reflecting abundant organic inputs [61,62]. Conversely, WE soils had lower nutrients and microbial diversity due to limited organic matter and unstable moisture conditions [63,64,65]. MF and CF soils, especially deeper layers, showed lower nutrient content and microbial diversity, linked to slower nutrient cycling and reduced biomass [66,67]. Soil nutrients and microbial diversity influence each other, jointly affecting nutrient cycling and soil health. Wetlands’ rich organic matter and stable moisture promote diverse microbial communities, enhancing nutrient accumulation, while forest ecosystems exhibit lower microbial diversity and nutrient availability due to limited inputs and slower decomposition [68,69].

### 4.2. Environmental Factors Influencing Soil Microbial Community Structure

Terrain, nutrient cycling, global change factors, plant litter, land use, soil organic matter, soil depth, and microbial structure significantly influence plant and microbial diversity in forest–grassland ecotones [70]. Soil microorganisms are key indicators of soil health and ecosystem stability [71]. Analysis of microbial phyla across ecotones showed similar overall compositions with notable differences in relative abundance. Actinobacteria, dominant in mixed forests (MF) and coniferous forests (CF), specialize in decomposing organic matter [72]. Acidobacteriota, abundant in wetland ecotones (WE), thrive in acidic environments typical of these soils. Proteobacteria dominated natural wetlands (NW), reflecting their critical roles in nutrient cycling, particularly nitrogen fixation, in moisture-rich habitats [73]. Microbial compositions showed minimal differences between soil layers (0–15 cm and 15–30 cm), likely due to similar environmental conditions within this depth range [74,75,76].

Microbial communities were significantly influenced by ecological factors, including soil pH, organic matter, moisture, and temperature [77]. Acidic soils favored Acidobacteriota, nutrient-rich wetlands supported Proteobacteria, and slower decomposition in forests promoted Actinobacteria growth. PCA and NMDS analyses confirmed distinct microbial communities across ecotones, notably between NW and WE (Figure 3a,b). Soil depth had limited influence, although differences between layers were driven by factors like potassium (AK), bulk density (BD), soil organic carbon (SOC), available phosphorus (AP), and total nitrogen (TN) (Figure 4). Potassium and BD strongly influenced MF and CF microbial structures, while SOC and AP shaped WE communities. In NW, deeper soil microbial diversity negatively correlated with TN and TP, suggesting nutrient limitations.

Bacterial phyla abundances correlated significantly with soil properties. Proteobacteria abundance positively correlated with TN and AK, aligning with their nitrogen cycling roles [78,79,80]. Bacteroidota abundance positively correlated with TN, pH, SOC, and AK but negatively with total potassium (TK), highlighting their preference for organic-rich soils and potential suppression by excess potassium [81,82,83]. Desulfobacterota correlated positively with SOC, AP, TN, and available nitrogen (AN), reflecting their importance in nutrient and sulfur cycling in organic-rich environments [84,85,86]. Acidobacteriota and Verrucomicrobiota negatively correlated with AK, indicating their adaptation to lower nutrient conditions [87,88,89]. Actinobacteriota and Gemmatimonadota negatively correlated with SOC and AP, suggesting adaptation to nutrient-poor, oligotrophic conditions [90,91,92].

### 4.3. Functional Prediction Analysis of Soil Bacterial Communities in Forest–Wetland Ecotones

The results of this study reveal significant differences in the functional genes of soil bacterial communities across ecotones and soil depths (Figure 6). Interactions, such as bacterial competition and symbiosis, influence metabolic pathways and the expression of specific genes [93]. Niche differentiation leads to the enrichment of certain functional genes due to variations in energy acquisition and resource utilization [94]. Soil properties, including nutrient availability, oxygen levels, and organic matter content, directly shape microbial community structure and ecosystem function [95]. Higher abundances of chemotrophic and aerobic chemotrophic bacteria were found in MF and CF soils, particularly at 15 and 30 cm depths (MF_15_, MF_30_, CF_15_, CF_30_). These ecosystems provide favorable conditions for bacteria utilizing chemical compounds and oxygen for energy, consistent with temperate and boreal forest ecosystems [96]. These bacteria are vital for nutrient cycling processes, such as nitrogen oxidation and sulfur reduction [97]. Enhanced oxygen diffusion and higher organic carbon content in MF and CF soils likely support these microbial communities, fostering a diverse and stable environment [98,99]. The elevated abundance of animal parasitic or symbiotic bacteria in MF15 suggests close interactions with plant roots, reflecting conditions favorable for mutualistic and parasitic relationships [100,101]. Their decreased presence at deeper soil layers highlights variations in nutrient availability, moisture, and plant–root interactions [35]. Fermentation-related bacteria were most abundant in WE and NW, indicating the dominance of anaerobic processes typical of wetland ecosystems with low oxygen and high organic matter [102,103,104,105]. These bacteria significantly contribute to carbon cycling and nutrient recycling, supporting ecosystem sustainability [62,106]. BugBase phenotypic prediction identified significant variations in bacterial abundance and functional traits across different ecotones and depths (Figure 6b). NW_30_ soils exhibited lower biofilm formation, likely due to reduced nutrient availability and oxygen levels at greater depths [107,108]. Gram-negative bacteria dominated WE and NW soils, thriving under fluctuating wetland conditions and playing key roles in nutrient cycling [109,110]. MF_15_ soils had the highest abundance of stress-tolerant and aerobic bacteria, attributed to favorable oxygen conditions and organic content that support microbial resilience and aerobic respiration [111,112]. Metal-tolerant bacteria were most abundant in CF_30_ and NW_30_, indicating these soils’ metal-rich environments potentially due to natural concentrations or contamination [113,114,115]. These specialized bacteria contribute significantly to bioremediation and nutrient cycling under metal stress conditions [116].

## 5. Conclusions

This study provides valuable insights into the microbial community structure and functional traits in soils across different forest–wetland ecotones. Our results show significant differences in bacterial abundance and community composition, which are closely related to soil properties, such as nutrient availability, organic carbon, and potassium content. Soil type plays a crucial role in shaping microbial community structure, with Proteobacteria, Actinobacteria, and Acidobacteriota exhibiting different abundances in different ecological environments. Notably, the high abundance of Proteobacteria in natural wetlands and the dominance of Actinobacteria in mixed and coniferous forest soils reflect their key roles in nutrient cycling, organic matter degradation, and soil health. Additionally, environmental factors, such as pH, organic carbon, and nitrogen content, significantly influence bacterial community composition, highlighting the adaptive strategies of different bacterial groups to their respective ecological niches. Interestingly, although microbial communities in different ecotones show some structural differences, the composition of microbial communities between different soil layers within the same soil type showed little variation, suggesting that soil depth has a limited impact on microbial diversity. This phenomenon may be related to the similar environmental conditions between surface and deep soils, especially in humid environments, where differences in moisture and nutrient supply across soil depths are minimal, leading to a high similarity of microbial communities between different soil layers. Functional prediction analysis indicates a significant impact on bacterial community functions across different forest–wetland ecotone soils. Different microbial groups adapt to specific ecological niches and participate in key ecological processes, such as nutrient cycling and organic matter degradation. These processes include biofilm formation, metal tolerance, and stress tolerance, revealing the key roles of soil depth and ecological environment in shaping microbial community composition. The lower abundance of biofilm-forming bacteria in deep wetland soils and the higher abundance of metal-tolerant bacteria in soils with elevated metal concentrations highlight their importance in bioremediation and nutrient cycling. Future research should focus on exploring the functional roles of specific bacterial groups in these ecotones, particularly their contributions to biogeochemical cycles. Further research into the interactions between microbial communities and their physical–chemical environments will help deepen our understanding of how ecological transitions between wetland and forest ecosystems influence microbial dynamics.

## Figures and Tables

**Table 1:** Vegetation types across the ecotone from mixed forest to natural wetland.

Ecotones Type	Abbreviation	Plant Composition	Plant Shannon Diversity Index
Mixed forest	MF	*Betula platyphylla* *Larix olgensis* *Picea jezoensis*	0.88 ± 0.03 a
Conifer forest	CF	*Larix olgensis* *Lonicera caerulea* *Spiraea salicifoliab*	0.57 ± 0.03 b
Wetland edge	WE	*Alnus sibirica* *Syringa reticulata* *Prunus padus* *Anemone dichotoma*	0.54 ± 0.04 b
Natural wetland	NW	*Carex schmidtii* *Deyeuxia angustifolia* *Sanguisorba tenuifolia* *Filipendula Palmata*	0.52 ± 0.02 b

Note: Plant Shannon diversity index (means ± standard); different lowercases represent significant difference at *p* < 0.05 level, tested with Duncan multiple comparisons.

**Table 2:** The physicochemical properties of soil in different forest–wetland ecotones.

Variables	MF_15_	MF_30_	CF_15_	CF_30_	WE_15_	WE_30_	NW_15_	NW_30_
pH	5.4 ± 0.0 b	5.3 ± 0.1 b	5.6 ± 0.1 ab	5.8 ± 0.1 ab	5.8 ± 0.1 ab	5.8 ± 0.2 ab	5.6 ± 0.3 ab	6.1 ± 0.4 a
SOC (g·kg^−1^)	51.3 ± 2.7 b	41.8 ± 0.6 c	35.7 ± 2.1 d	33.2 ± 0.4 d	50.1 ± 1.2 b	43.2 ± 1.5 c	59.8 ± 1.8 a	53.5 ± 1.9 b
BD (g·cm^−3^)	1.2 ± 0.1 a	1.5 ± 0.6 a	1.1 ± 0.17 a	1.3 ± 0.0 a	1.2 ± 0.0 a	1.4 ± 0.1 a	1.2 ± 0.1 a	1.4 ± 0.1 a
TN (g·kg^−1^)	4.3 ± 0.8 b	4.5 ± 0.1 b	3.1 ± 1.0 c	2.9 ± 0.1 c	3.3 ± 0.1 c	1.7 ± 0.3 d	6.7 ± 0.3 a	6.5 ± 0.0 a
AN (mg·kg^−1^)	122.7 ± 5.1 b	93.7 ± 11.6 b	64.3 ± 5.2 c	51.5 ± 2.5 c	108.4 ± 2.0 b	76.3 ± 4.8 c	168.9 ± 3.4 a	105.9 ± 2.8 b
TP (g·kg^−1^)	0.5 ± 0.0 a	0.4 ± 0.0 b	0.3 ± 0.0 c	0.2 ± 0.0 c	0.3 ± 0.0 c	0.2 ± 0.0 c	0.5 ± 0.0 a	0.4 ± 0.0 b
AP (mg·kg^−1^)	53.0 ± 0.4 d	47.1 ± 2.4 d	41.1 ± 2.4 e	31.7 ± 0.5 f	73.2 ± 2.1 b	68.2 ± 0.2 c	115.1 ± 9.2 a	105.7 ± 2.6 a
TK (g·kg^−1^)	7.1 ± 0.3 a	5.1 ± 0.3 b	2.4 ± 0.2 d	2.1 ± 0.3 d	4.0 ± 0.1 c	3.3 ± 0.7 c	5.4 ± 1.1 b	3.7 ± 1.2 c
AK (mg·kg^−1^)	164.7 ± 2.1 b	143.2 ± 1.9 c	152.5 ± 2.5 bc	148.7 ± 2.7 c	91.3 ± 5.2 d	75.2 ± 8.0 e	183.4 ± 11.2 a	156.4 ± 4.8 b

Note: values represented mean ± standard deviations (*n* = 3). Different letters stand for significant effects (*p* < 0.05). Abbreviations: pH, soil pH value; BD, soil bulk density; SOC, soil organic carbon; TN, total nitrogen; TP, total phosphorus; TK, total potassium; AN, available nitrogen; AP, available phosphorus; AK, available potassium; MF, mixed forest; CF, conifer forest; WE, wetland edge; NW, natural wetland. The symbols 15 and 30 for soil samples in the table represent soil depths ranging from 0–15 cm and 15–30 cm, respectively.

**Table 3:** The alpha diversity of soil bacteria in different forest–wetland ecotones.

Soil Sample	Sobs	Shannon	Ace	Chao1
MF_15_	1032.7 ± 101.2 d	5.5 ± 0.1 c	1139.7 ± 122.1 d	1149.4 ± 116.9 d
MF_30_	1081.3 ± 66.6 d	5.7 ± 0.1 c	1207.0 ± 63.2 d	1221.0 ± 74.8 d
CF_15_	1412.3 ± 62.3 bc	6.2 ± 0.1 b	1517.8 ± 49.8 b	1566.4 ± 22.6 b
CF_30_	1448.3 ± 18.2 b	6.2 ± 0.0 b	1543.5 ± 17.1 b	1573.0 ± 13.5 b
WE_15_	1276.7 ± 91.2 c	5.8 ± 0.0 c	1419.3 ± 94.3 bc	1465.3 ± 85.1 bc
WE_30_	1277.7 ± 27.7 c	5.8 ± 0.1 c	1420.3 ± 31.0 c	1457.7 ± 14.7 c
NW_15_	1611.7 ± 82.3 a	6.4 ± 0.0 a	1751.5 ± 68.1 a	1785.3 ± 58.5 a
NW_30_	1651.0 ± 33.1 a	6.4 ± 0.1 ab	1806.1 ± 25.7 a	1852.3 ± 35.7 a

Note: values represented mean ± standard deviations (*n* = 3). Different letters stand for significant effects (*p* < 0.05).

**Table 4:** Pearson’s correlation coefficients among soil physicochemical properties and the top 10 bacterial phyla measured in different forest–wetland ecotones.

Phylum	BD	TP	TK	TN	AK	pH	AN	SOC	AP
Actinobacteriota	0.111	−0.094	−0.132	−0.172	0.156	−0.296	−0.451	−0.585	−0.749
Acidobacteriota	−0.103	−0.135	0.171	−0.368	−0.525	0.047	0.010	−0.126	−0.044
Proteobacteria	−0.026	0.142	0.001	0.571	0.547	0.072	0.229	0.351	0.355
Chloroflexi	0.061	−0.100	0.163	−0.389	−0.354	−0.226	−0.192	−0.290	−0.397
Firmicutes	−0.091	−0.484	−0.741	−0.291	−0.038	0.306	−0.511	−0.361	−0.232
Gemmatimonadota	−0.049	−0.017	0.026	−0.130	0.307	−0.374	−0.366	−0.528	−0.727
Bacteroidota	−0.079	0.011	−0.279	0.424	0.213	0.441	0.262	0.463	0.654
Desulfobacterota	−0.174	0.195	0.016	0.406	0.226	0.305	0.492	0.701	0.853
Verrucomicrobiota	−0.031	0.025	0.239	−0.306	−0.548	−0.047	0.021	−0.155	−0.173
Myxococcota	−0.025	−0.375	−0.570	−0.087	0.257	0.135	−0.439	−0.431	−0.370

## Data Availability

The datasets generated during and/or analyzed during the current study are available from the corresponding author upon reasonable request.
